# The Potential Mechanisms of Banxia Xiexin Decoction in Treating Chronic Colitis: Insights from UPLC-Q-TOF-MS/MS and Network Pharmacology Studies

**DOI:** 10.2174/0113862073350796250305225907

**Published:** 2025-07-14

**Authors:** Xinyao Pan, Ruyun Zhang, Mengyuan Wang, Chunjuan Yang, Jinhui Wang, Chunli Gan

**Affiliations:** 1 Department of Medicinal Chemistry and Natural Medicine Chemistry, College of Pharmacy, Harbin Medical University, Harbin, 150081, P.R. China;; 2 Department of Pharmaceutical Analysis and Analytical Chemistry, College of Pharmacy, Harbin Medical University, Harbin, 150081, P.R. China

**Keywords:** Banxia xiexin decoction, chronic colitis, inflammation, UPLC-Q-TOF-MS/MS, network pharmacology, molecular docking

## Abstract

**Introduction:**

Banxia Xiexin Decoction (BXD), traditionally used for gastrointestinal disorders like Chronic Colitis (CC), exerts anti-inflammatory, antibacterial, and intestinal flora-regulating effects. However, CC’s pathogenesis remains unclear, necessitating further research into BXD’s machanism.

**Methods:**

Active BXD components were identified *via* UPLC-Q-TOF-MS/MS. Databases (TCMSP, HERB, GeneCards,DisGeNET,STRING) were used to identify compound/disease targets. Cytoscape 3.9.1 constructed protein-protein interaction networks, and DAVID database was used for GO and KEGG enrichment analysis of core genes. Finally, PyRx, AutoDockTools and PyMol were used for molecular docking, virtual computation, and visualization analyses of core components and key targets.

**Results:**

UPLC-Q-TOF-MS/MS detected 482 BXD components, with 165 active ingredients, including quercetin, kaempferol, baicalein, etc. There were 283 targets related to BXD's treatment of CC, of which the core targets included AKT1, IL-6, TP53, ALB, etc. GO enrichment analysis yielded relevant entries including molecular function 60 entries, 257 entries of biological processes, and 31 entries of cellular composition, and KEGG enrichment analysis identified 150 entries involving IL-17, TNF, PI3K-Akt, and other pathways. The molecular docking results demonstrated that the core components exhibited better binding activities with the key targets.

**Discussion:**

Quercetin, kaempferol, baicalein, and naringenin, the main active ingredients in BXD, may play roles in anti-inflammatory, antimicrobial, and regulating intestinal microbiota to achieve the therapeutic purpose of CC treatment by mediating the targets of AKTl, IL-6, TP53, and ALB, and regulating the signaling pathways of IL-17, TNF, and PI3K-Akt.

**Conclusion:**

BXD’s active components alleviate CC through multi-target and multi-pathway regulation, providing a mechanistic foundation for clinical application.

## 1 INTRODUCTION

The issue of "inflammation and cancer transformation" [1, 2] has drawn widespread attention both at home and abroad and has become the focus of numerous scholars. Chronic Colitis (CC) is taken as an illustrative example, its prevalence is increasing rapidly in China, and the affected population is showing a trend of getting younger. It is a chronic intestinal disease characterized by recurrent diarrhea, abdominal pain, mucus, pus and blood in stools, along with acute and severe symptoms. In traditional Chinese medicine (TCM), it is associated with "*xie xie*" (diarrhea), "*chang du*" (intestinal toxins), and "*pi zheng*" (epigastric fullness) [3]. The direct etiology of the disease remains undetermined. Western medicine posits that it may be closely related to factors such as heredity, infection and diet. Conversely, traditional Chinese medicine considers that it might be closely associated with constitutional insufficiency and internal injuries caused by seven emotions [4]. Western medicine commonly employs immunosuppressants, glucocorticoids like dexamethasone, and the anti-inflammatory drug mesalazine for treatment purposes [5]. However, due to the protracted course of the disease, the condition is recalcitrant and prone to recurrence. What is more, the long-term use of chemotherapeutic agents entails numerous side effects and is liable to induce drug resistance. consequently resulting in an insignificant long-term efficacy. In the long run, there is a risk of "inflammation and cancer transformation", which exacerbates patients’ psychological burden regarding the fear of cancer [1]. Banxia Xiexin Decoction (BXD) originating from “Zhang Zhongjing's *Treatise on Cold-induced and Miscellaneous Disease*”. It consists of 7 herbs: *Pinellia Ternata* (Banxia, BX), *Scutellariae Radix* (Huangqin, HQ), *Coptidis Rhizoma* (Huanglian, HL), *Zingiberis Rhizoma* (Ganjiang, GJ), *licorice* (Gancao, GC), *Panax Ginseng*. (Renshen, RS) and *Jujubae Fructus* (Dazao, DZ). In the formula, BX serves to disperse nodules, eliminate epigastric fullness, dry dampness, and resolve phlegm, functioning as the principal herb. GJ warms the middle and disperses cold, while HQ and HL, both bitter and cold in nature, clear heat, acting as deputy herbs. RS and DZ strengthen the spleen and replenish qi, functioning as assistant herbs. GC strengthens the spleen and harmonizes the middle, serving as both an assistant and envoy herb. Together, they achieve the effects of acrid dispersal and bitter descent, combining attack and tonification. This formula balances cold and heat, regulates qi, and alleviates epigastric fullness, making it highly suitable for addressing the "*pi zheng*" (epigastric fullness) associated with (CC) [6]. Clinical applications of BXD in patients with chronic colitis have demonstrated low adverse effects, good long-term efficacy, and an significant improvement in the patients’ quality of life [6]. Nevertheless, its mechanism of action has not been fully elucidated.

In this study, we employed UPLC-Q-TOF-MS/MS to determine the composition of BXD. Subsequently, based on the main active components within BXD and the pathogenic targets of CC, we analyzed the interactions among "multi-components, multi-targets, and multi-pathways" within the pharmacological network nodes. Through this approach, our aim was to explore the potential mechanism underlying BXD’s efficacy in treating CC and further validate it at the molecular level by utilizing molecular docking technology. This study endeavors to elucidate the scientific connotation of BXD from a modern scientific perspective, thereby providing a valuable reference for expanding its clinical application.

## 2 MATERIALS AND METHODS

### 2.1 Databases, Platforms and Software

The databases, platforms and software adopted in this study are shown in Table **1**.

### 2.2 Technological Route

The technical route of this study is depicted in Fig. (**1**).

### 2.3 Experimental Herbs

The description of BXD in the *Treatise on Cold-induced and Miscellaneous Disease* is as follows, "half a liter of *Pinellia Ternata* (Banxia, BX), three taels each of *Scutellariae Radix* (Huangqin, HQ), *Panax Ginseng*. (Renshen, RS), *Zingiberis Rhizoma* (Ganjiang, GJ), and roasted *licorice* (Gancao, GC), one tael of *Coptidis Rhizoma* (Huanglian, HL), and twelve *Jujubae Fructus* (Dazao, DZ)." This information was supplemented with relevant literature [6-8]. Based on this, the prescription ratio of BXD was calculated as BX: RS: HL: HQ: GC: GJ: DZ= 4:3:1:3:3:3:1. The details regarding the herbs, herb lots, origins, and weighing qualities of BXD raw herbs, are presented in Table **2** below. It should be noted that the quantity of *Jujubae Fructus* (DZ) was counted in terms of the number, specifically 12 in pieces. All the herbs were provided by the Pharmaceutical Factory of Harbin Medical University, located in Heilongjiang Province, China.

### 2.4 Reagents

The reagents methanol (HPLC grade), acetonitrile (HPLC grade), formic acid (HPLC grade) were purchased from Beijing Mile High Technology Co.

### 2.5 Sample Preparation

The BXD raw herbs were weighed precisely according to the preset proportion. Subsequently, they were soaked in 2 L of pure water for 1 hour. After that, the soaked herbs were boiled and decocted for 40 minutes, then decocted at 80°C for an additional 20 minutes, and extracted by heating and refluxing for a total of 1 hour. Subsequently, the filtrate was collected through filtration. Then 1.6 L of pure water was added to the residue of the filtrate. The mixture was boiled and decocted for 30 minutes before being further decocted at 80°C for 20 minutes, with the entire heating and reflux extraction process lasting for 50 minutes. Subsequently, the filtrate obtained from the previous step was collected *via* filtration, Then, the two filtrates were combined and concentrated by evaporation until the volume was reduced to half of the total weight of the herbs, thus obtaining the aqueous extract of BXD. Next, 1000 μL of the BXD aqueous extract was pipetted, and 1000 μL of methanol was added to it. The mixture was vortexed for 30 s and then centrifuged at 15000 r/min for 15 min at 4°C. Subsequently, the supernatant was collected and filtered through a 0.22 μm microporous filter membrane, Finally, it was placed in the refrigerator at 4°C for subsequent analysis.

### 2.6 Determination of Chemical Constituents in Banxia Xiexin Decoction by UPLC-Q-TOF-MS/MS Technique

#### 2.6.1 Sample solution preparation

First, 1000 μL of the BXD aqueous extract was pipetted, and then 1000 μL of methanol, was vortexed for 30 s. After that, it was centrifuged at 15000 r/min for 15 min at 4°C. Next, the supernatant was collected and filtered through a 0.22 μm microporous membrane. Finally, the filtered sample was placed in the refrigerator at 4°C for subsequent analysis [9].

#### 2.6.2 UPLC-Q-TOF-MS/MS conditions

Chromatographic separation was carried out employing a Waters UPLC BEH C18 column (2.1 × 50 mm × 1.7 μm). The mobile phase was composed of solvent A (ultrapure water containing 0.1% formic acid) and solvent B (methanol). Gradient elution was conducted with this system, and the specific gradient elution conditions are presented in Table **3** below. The flow rate was set at 0.2 mL/min, the sample compartment temperature was maintained at 4°C, and the column temperature was maintained at 45°C, The injection volume was set to 1 μL.

The samples were analyzed using an electrospray ionization source (ESI). The data were collected in MS^E^ mode, with the mass spectral data being acquired simultaneously in both positive and negative ion modes as well as in resolution modes. For real-time calibration of the mass number, the instrument was calibrated using leucine encephalin (LE) standards, where the m/z values were set at 556.2771 for positive ions and 554.2615 for negative ion mode, at a flow rate of 10 μL/min. The MS data acquisition parameters are shown in Table **4**. Ultra-high performance liquid chromatography with quadrupole time-of-flight mass spectrometry (UPLC-Q-TOF/MS) was employed to compare the theoretical values with the measured values, to qualitatively identify the chemical constituents within the decoction of BXD. Subsequently, the Base Peak Ion (BPI) was obtained in the positive-negative ion mode, as depicted in Fig. (**2**). The MS^E^ data were collected through the application of the MassLynx V4.1 software. Based on the literature and the DPN 24.2 Dictionary of Natural Products, a database of the compounds was established. Subsequently, a table was created to import the data into the UNIFI software for peak identification and peak fingerprinting [10-12].

### 2.7 Collection of Banxia Xiexin Decoction Active Ingredients and Related Targets

Based on the results obtained from UPLC-Q-TOF-MS/MS, the screening procedures for active ingredients and targets were initiated. The active ingredients of BXD were retrieved from TCMSP [13] and HERB [14] databases and the thresholds were defined as oral bioavailability (OB) ≥30% and drug-likeness (DL) ≥0.18 for screening the active ingredients. Subsequently, by employing STRING, an online protein-protein interaction (PPI) network analysis database [15, 16], the screened targets were converted into corresponding Gene Symbols. Regarding the data that could not be found in the STRING database, Gene symbols were obtained by applying the entries acquired from the UniProt [16] database screening.

### 2.8 Access to Chronic Colitis Disease Targets

The keyword "chronic colitis" was set, and searched were conducted in the DisGeNET [17] and GeneCards [18] databases. In the GeneCards database, the Relevance Score indicates the relevance of the target to the disease. Specifically, the higher the value of the Relevance Score, the stronger the link between the target and CC. Targets with high relevance were selected based on the Relevance Score [19, 20]. Subsequently, the disease targets were standardized *via* the UniProt database, and the target set of CC was obtained by deleting duplicates.

### 2.9 Constructing Herb-Ingredient-Target Maps

Based on the active ingredients of BXD and their corresponding targets, Cytoscape 3.9.1 [16] software was employed to construct a "Herb-Ingredient-Target" network. Subsequently, the topological analysis of the network was carried out using the “Network Analyzer” plugin.

### 2.10 Building PPI Networks

Based on the obtained targets corresponding to the active ingredients and the targets related to CC disease, the visualization of the intersection between the ingredient targets and CC targets was performed using the Venn diagram online tool available in Bioinformatics [21]. These intersecting genes represent potential targets of BXD for CC therapy. Subsequently, the targets at the intersection of the two were extracted, and these intersecting targets were imported into the STRING database for PPI network analysis. The analysis mode was set as “Multiple proteins”, the species was specified as "Homo sapiens", the minimum required interaction score was set to "medium confidence (0.400)", and the isolated proteins were configured to be hidden. Thereafter, the obtained TSV file was imported into Cytoscape 3.9.1 software for visualization purposes, enabling the drawing of the PPI network diagram. Subsequently, the "Network Analyzer" was utilized to conduct network topology analysis. In this analysis, the core targets were identified based on these parameters to deeply analyze the properties of the nodes within the interaction network, Specifically, the filtering was carried out through the Betweenness Centrality (BC), Closeness Centrality (CC), and Degree Centrality (DC) [19, 22].

### 2.11 GO Enrichment and KEGG Pathway Analysis

GO and KEGG pathway enrichment analyses were performed in DAVID [23-25] by inputting the "Gene symbol" of the two intersecting targets, with "Homo sapiens" set as the species set. Subsequently, the results of GO analysis were visualized as bar graphs accompanied by bubble plots using Bioinformatics tools, Gene ID conversion was carried out using the bioDBnet [26] database, in the form of a Sankey Diagram along with bubble plots. The *p*-value determines the outcome of the correlation test [22, 27], and the top ten items in each category were ranked in descending order according to the -Log_10_P value, followed by the plotting of the bubble chart. The KEGG signaling pathways were visualized using R version 4.40.

### 2.12 Construction and Topology Analysis of "Active Ingredient-Target-Pathway" Network

To better illustrate the relationship among components, targets, and pathways, an " Ingredient-Target-Pathway" network diagram was constructed in Cytoscape 3.9.1 software, and topological analysis was conducted. Subsequently, the core components were identified using BC, CC, and DC analyses [28].

### 2.13 Molecular docking

Molecular docking is a commonly employed methodology in drug discovery and a significant drug design technique for investigating receptor and ligand interactions and recognition. It is a theoretical simulation method used to study intermolecular interactions and predict their binding modes and affinities [24, 29]. Consequently, the molecular docking methodology was adopted to examine whether the core component of BXD identified through network pharmacology binds to the core protein. The specific process is described below:

#### 2.13.1 Preparation of Protein Receptor Files

Based on the results of the preliminary network pharmacology screening, the 3D structure of the target protein was retrieved from the protein database (PDB) [30, 31] and downloaded the PDB format file. The protein was then processed in AutoDockTools 1.5.6 [32] with the assistance of PyMol software to remove the water molecule along with the self-contained ligand. The specific operations are as follows: the water was removed, hydrogen atoms were added in its place, the protein was designated as a receptor, and the structure was saved as a PDBQT protein receptor file.

#### 2.13.2 Preparation of Small Molecule Ligand Files

Based on the molecule MOL ID, the corresponding chemical name of its chemical structure was retrieved from the TCMSP database. Then PubChem [33] was utilized to obtain the ligand SDF format file. Thereafter, the SDF format of the ligand was converted into the MOL2 format using OpenBabelGUI [34]. AutoDockTools 1.5.6 was employed to configure the drug as a ligand by removing water molecules and adding hydrogen atoms. Finally, the file was exported as PDBQT format small molecule ligand file.

#### 2.13.3 Selection of the Docking Object and Calculation of the Binding Energy

Import the receptor and ligand in PDBQT format into PyRx [35] software. Upon successful import, the interface will display the ligand and receptor. Subsequently, set the receptor and ligand, and the corresponding ligand and receptor will be shown in the AutoDock column. Select "Vina Wizard", then click "Start". Next, click "Forward" and modify the parameters as required, followed by selecting "Maximize". Finally, click "Forward". Through these steps, the display of molecular docking binding energies can be achieved in advance. Analyze the binding energy data calculated by RyRx software, and then filter the ligands and receptors corresponding to molecular docking with binding energies less than -8 Kcal/mol. Subsequently, reconfirm the molecular docking using AutoDockTools 1.5.6. In this study, AutoDockTools 1.5.6 was adopted as the reference standard. It was considered that binding energies less than -4.25 kcal/mol (1 kcal ≈ 4.18585 kJ) suggested some binding activity between ligand and receptor, those less than -5.0 kcal/mol demonstrated good binding activity, and less than -7.0 kcal/mol exhibited strong binding activity [36].

The configured PDB format file was exported. Receptors and ligands with hydrogen bonding, were visualized directly using PyMol software. However, receptors and ligands without hydrogen bonding, would be imported into AutoDockTools 1.5.6 in PDB format using PLIP [37]. This tool is capable of identifying and displaying non-covalent interactions between biomolecules and their ligands. Subsequently, the PSE format file was downloaded and imported into PyMol software for visualization purposes (**Supplementary material**).

## 3 RESULTS

### 3.1 Chemical Composition Analysis of Banxia Xiexin Decoction Decoction Solution

482 compounds were successfully detected without categorization. In the negative ion mode: 108 species of BX, 89 species of DZ, 239 species of GC, 49 species of GJ, 62 species of HL, 150 species of HQ, and 86 species of RS, and in the positive ion mode: 153 species of BX, 222 species of DZ, 200 species of GC, 159 species of GJ, 125 species of HL, 152 species of HQ, and 220 species of RS.

### 3.2 Banxia Xiexin Decoction Chemical Composition and Target Screening Results

The 165 Banxia Xiexin Decoction active ingredients were screened. Among them, 12 compounds were found to be present in two or more herbs (see Table **5**). The combined database yielded 283 gene names for each target.

### 3.3 Results of Construction and Analysis of Herb-Ingredient-Target Network

As can be seen in Fig. (**3**), there are 455 nodes and 3557 edges. Nodes denote drugs and targets and edges denote connections between them. Among them, there are 7 circular nodes, which are DZ, BX, GJ, HL, RS, HQ, and GC in clockwise order from left to right. In addition, 12 hexagonal nodes represent the common components of the 7 herbal medicines, and the IDs displayed by the hexagonal nodes are shown in Table **5**. In addition, the unique components of each of the 7 herbal medicines were represented by 153 square nodes.

### 3.4 PPI Network Construction and Analysis

By comparing TCM active ingredient targets and CC disease targets, 159 targets were identified as their intersecting genes, as shown in Fig. (**4**). Subsequently, these intersecting genes were involved in 159 nodes and 4406 edges. In this network, nodes represent the roles of the targets while edges represent the connections between the nodes. The PPI network is centered on the nodes of the core genes (AKT1, IL-6, TP53, ALB, IL1B, JUN, and CASP3), which exhibit high node connectivity values. The nodes in the graph represent targets that cross each other, and the higher the degree of centrality value, the larger the area of the graph. 14 core targets were filtered out (see Table **6**), using BC greater than the median 0.00215, CC greater than the median 0.59176, and DC greater than twice the median 106) as conditions.

### 3.5 Enrichment Analysis Results

DAVID was utilized to conduct GO function enrichment analysis and KEGG pathway enrichment analysis on the 159 targets intersected by the two. The screening conditions were set with a false discovery rate (FDR) of less than 0.01. Consequently, 348 GO entries were obtained, including 60 entries of molecular function (MF), 257 entries of biological process (BP), 31 entries of cellular component (CC), and 150 entries of KEGG pathway, shown in Fig. (**5A**).

The color shades of the bubble diagrams in the figure represent the -Log_10_ *P* values of the corresponding entries, and the horizontal axis of the bubble diagrams, denoted as gene ratio (%), refers to the percentage of the ratio between the number of genes in the entries (count) and the number of intersecting genes among the 159 genes involved in the enrichment. Based on the comprehensive analysis of Fig. (**5B**, **5C**), the GO enrichment results indicated that the targets regulated by the active components of BXD were located in cellular components such as the cytosol, nucleus, extracellular space, extracellular region, and macromolecular complex. These targets were also involved in biological processes like the positive regulation of gene expression, positive regulation of transcription from RNA polymerase II promoter, positive regulation of transcription, DNA-templated, and others. Moreever, they possessed molecular functions, including enzyme binding, identical protein binding, protein binding, and protein homodimerization activity.

Furthermore, BXD active ingredients regulate the targets within cellular components such as cytosol, nucleus, extracellular space, extracellular region, macromolecular complex The results of the KEGG enrichment analysis on the key genes were obtained (as shown in Table **7**).

The top 20 entries were visualized by sorting each category in descending order according to the -Log_10_P value (as shown in Fig. (**6A**, **6B**)). Using Bioinformatics tools, Sankey diagrams were constructed based on the connectivity between target genes and enrichment pathways (as shown in Fig. (**6C**, **6D**)). As revealed by the KEGG enrichment analysis, the targets were mainly enriched in pathways related to cancer, lipid and atherosclerosis, as well as signaling pathways such as IL-17, PI3K- Akt and TNF. Among the top 20 pathways enriched, the core targets AKT1, IL-6, TP53, JUN and CASP3 were involved in 17, 11, 14, 12 and 12 pathways, respectively. The high enrichment of IL-17, PI3K-Akt and TNF signaling pathways in the GO and KEGG databases suggests that BXD may play a role in the treatment of CC through these three pathways. We visualized the three KEGG pathways using the "pathway view" in R (Fig. (**7**)).

### 3.6 Construction and Topology Analysis of Ingredient-Target-Pathway Network

The analysis was carried out using the “Analyze Network” function within the “Tools” menu of the Cytoscape software. After exporting the data, the BC and CC were retained to five decimal places, with the condition that the BC was greater than the median of 0.00068, the CC was greater than the median value of 0.39150, and the DC was greater than 25. Subsequently, seven core targets were screened out, as shown in Table **8**.

The 165 components, 159 targets, 10 signaling pathways, and 1 disease of BXD obtained from the above screening were constructed into an "Ingredient-Target-Pathway" network, and the labels on the component nodes were self-written IDs corresponding to the MOL IDs in the TCMSP database [28], resulting in a network of 342 nodes and 2540 edges in the network. The drug active ingredients are represented by color nodes corresponding to each of the seven herbs, the target of action is indicated by a purple node, and the pathway is presented by a light red node, as shown in Fig. (**8**).

### 3.7 Molecular Docking Validation

The first 7 core components were selected from the "Ingredient-Target-Pathway" network to serve as small molecule ligands (see Table **7**). Meanwhile, 14 core targets were identified from the PPI network of CC targets for BXD treatment (see Table **5**), and all of these 14 were utilized as large molecule receptors. Molecular docking between large molecule receptors and small molecule ligands was conducted, with small molecule ligands being baicalein, kaempferol, wogonin, naringenin, quercetin, beta-sitosterol, and Stigmasterol. And the target proteins for docking being AKT1, ALB, BCL2, CASP3, EGFR, ESR1, HIFIA, 1L1B, IL-6, JUN, MMP9, PTGS2, TP53, STAT3. (Fig. **9A**, **B**) shows the docking binding energy results from the Vina algorithm of the PyRx software combined with selective docking with AutoDockTools 1.5.6. Generally, if a ligand binds better to the receptor protein, the smaller the molecular docking fit value and the higher the likelihood of interaction will be. Moreover, usually, the lower the binding energy of the ligand to the receptor, the more stable the bound conformation. There were 12 groups with binding energies below -5.0 Kcal/mol. The 3D binding models of each target and component could be observed in the molecular docking predictions (as shown in Table **9**). As depicted in Fig. (**10**), the purple portion of the 5A amino acid residue pocket represents the site of connection *via* hydrogen bonding, The compound-target protein docking diagrams demonstrate that compounds and target proteins are capable of forming intermolecular forces such as hydrogen bonding, which further validates the credibility of the screening results. Beta-sitosterol, baicalein, kaempferol, naringenin, wogonin, and quercetin， these ligands are available through hydrogen bonding with receptor proteins in the active pocket with hydrogen bonding. Stigmasterol can bind to 7NH4 in the PDB database *via* hydrogen bonding but exhibits only hydrophobic interactions with 1H4K and 4H1Q receptors in the PDB database without hydrogen bonding (as shown in Fig. (**10K**, **10L**)). Based on these findings, we predicted that all of these compounds may play a crucial role in the treatment of CC.

## 4 DISCUSSION

Initially, a compositional screening of BXD was conducted to identify 165 active ingredients. Among them, the main chemical components include quercetin, β-sitosterol, kaempferol, baicalin, stigmasterol, wogonin, and naringenin, These components can work in combination with other active ingredients to act on multiple targets within the network. Among the predicted active ingredients, quercetin, baicalin and naringin have demonstrated relatively better therapeutic effects on CC. Quercetin, naringenin and baicalein belong to the flavonoid family. Relevant studies have revealed that flavonoids exert several effects as follows: (1) Protection of the colon *via* antioxidant effects [36]. Flavonoids possess the ability to scavenge free radicals, thereby functioning as antioxidants. This antioxidant property plays a crucial role in safeguarding the colon from oxidative damage, which is often associated with various pathological processes in chronic colitis. (2) Protection of the colon through anti-inflammatory effects [36]. In patients with CC, intestinal damage occurs, leading to increased intestinal permeability. Metabolites produced by intestinal microorganisms and further metabolized can inflict certain harm on the liver. Also, intestinal inflammation can cause complications such as depression. Flavonoids not only interfere with the activity of pro-inflammatory proteins and modulate the intensity of inflammation but also possess immunomodulatory properties. Specifically, they can inhibit T lymphocytes and reduce the activity of inflammatory factors [38, 39]. Baicalein, in particular, inhibits the expression of cyclooxygenase-2 (COX-2) and inducible nitric oxide synthase (iNOS). It can also attenuate the activity and phosphorylation of inhibitor of κB kinase β(IKKβ) and subsequently facilitate the degradation of the inhibitor of κBα(IκBα) [40]. In addition, baicalein can ameliorate colitis and inhibit COX-2 exhibiting anti-inflammatory activity by improving the intestinal epithelial barrier via the aryl hydrocarbon receptor (AhR)/interleukin-3(IL-3) pathway in type 2 innate lymphoid cells(ILC22s) [40-42]. Moreover, baicalein inhibits p65 phosphorylation and nuclear translocation, reduces the DNA binding activity of nuclear factor-κB (NF-κB), inhibits signal transducer, and activates transcription factor 3 (STAT3) phosphorylation and cell cycle protein D1 expression [40-43]. It also exhibits anticancer activity in both *in vitro* and *in vivo* models of colorectal cancer through the AKT pathway [43-45]. According to a study by Zhong *et al* [40], baicalein significantly alleviated the severity of dextran sulfate sodium (DSS)-induced colitis. (3) Protecting the gut by modulating gut microbes [31]. Quercetin has been shown to increase the abundance of probiotic bacteria, while naringenin can decrease the relative abundance of potentially pathogenic bacteria [46], which consequently helps in ameliorating inflammation. A related study [47] demonstrated that naringenin was capable of regulating intestinal permeability to inhibit the development of colitis. Related reviews have found that quercetin may carry out immune organ protection by reducing inflammation and oxidative stress, thereby improving the symptoms of CC [48, 49]. (4) Impeding "inflammation and cancer transformation" [1]. Naringenin, one of the preserved flavonoid glycosides in grapefruit, has been reported to possess anticancer activity. Activating transcription factor 3 (ATF3) is associated with apoptosis in human colon cancer cells [50]. The results of previous studies have indicated that naringenin has a strong chemopreventive potential against 1, 2-dimethylhydrazine (DMH)-induced colon carcinogenesis. However, further studies are required to elucidate the exact mechanism of action of naringenin. Additionally, wogonin is also a potential anticancer candidate [51, 52]. The results of the chronic colitis (CC) disease target analysis revealed that CC and BXD share 159 common targets. These targets are regarded as potential targets for BXD in the treatment of chronic colitis.

The results of the PPI network revealed the following targets: Akt1, IL-6, TP53, ALB, IL1B, JUN, Casp3, EGFR, mmp9, ptgs2, stat3, bcl2, esr1, and hif1a, focusing on the core targets such as STAT3, MMP9, TPGS2, IL-6 and IL-17. As a convergence point of numerous oncogenic signaling pathways, STAT3 is central to signal transduction and regulation of anti-tumor immune responses. STAT3 is widely over-activated in cancer and non-cancer cells in the tumor ecosystem and plays a crucial role in suppressing the expression of key immune activation regulators and promoting the production of immune-suppressive factors IL-6 [53]. STAT3 has been shown to play a key role in colon carcinogenesis and development and is considered to be a major downstream effector driven by IL-6 [54]. Matrix metalloproteinase 9 (MMP9) is a zinc-dependent endopeptidase that exerts its inhibitory effects on colon cancer through the "MMP9-Notch1-ARF-p53 axis" pathway, and MMP9 expression is strongly correlated with reduced ROS levels, decreased DNA damage, and up-regulation of mismatch repair pathways [55, 56]. Data from a series of studies have revealed that MMP9 maintains epithelial mucosal integrity by activating the EGFR-Sp1 signaling pathway, and can act as a tumor suppressor in colon cancer [55, 57], and is a notable clinical marker in clinical practice [58]. While TPGS2 is one of the biomarkers, it is aberrantly expressed in most cancer tissues, especially colon cancer, and may be associated with immune cell infiltration in the tumor microenvironment [59]. It has been found that wogonin completely inhibits the production and expression of inflammatory mediators, including IL-6 and COX-2 [20]. IL-6 is the most central inflammatory factor bridging inflammation and tumors [60], which has been demonstrated in the literature [61]. IL-6, as an activator of STAT3, induces STAT3 activation and promotes downstream signaling, which in turn is involved in colon cancer progression. Quercetin was found to alleviate dextran sulfate sodium (DSS)-induced colitis by enhancing intestinal integrity and hepatic antioxidant capacity, modulating ERK1/2-FKBP and RXR-STAT3 pathways, increasing serum glutathione levels, and inhibiting oxidative stress in an H_2_O_2_-induced Caco-2 cell model [62].

To explore the mechanism of BXD treatment of CC, we performed GO analysis and KEGG enrichment analysis. The GO results indicated that biological processes involving positive regulation of gene expression, positive regulation of transcription from RNA polymerase II promoter, positive regulation of transcription, DNA-templated, and other biological processes were involved. KEGG enrichment analysis showed that BXD involved several signaling pathways in CC treatment, mainly including IL-17, PI3K-Akt, and TNF signaling pathways. The PI3K-AKT signaling pathway is highly enriched, and we hypothesized that this pathway may be the main signaling pathway for the treatment of CC. PI3K/Akt, as a common "astrocytic pathway" in digestive diseases, is closely related to cell proliferation, apoptosis, and angiogenesis, which have a great impact [63]. Clinical studies have confirmed that phosphorylated Akt (p-Akt) effectively enhances the phosphorylation of IjBa, thereby initiating NF-JB through activation of the PI3K/Akt signaling pathway [64, 65]. Activation of NF-JB promotes the secretion and expression of pro-inflammatory cytokines, leading to an imbalance in cytokine secretion, mucosal injury, inflammatory response, and exacerbation of CC symptoms. Studies have also confirmed that PI3K/Akt/mTOR signaling is one of the most commonly dysregulated signaling pathways in colon cancer patients, which plays a crucial role in tumorigenesis, progression, and therapeutic process, and it is essential in cell growth, metabolism, and so on [66, 67]. MicroRNA (Mir-223) can effectively upregulate the PI3K/Akt-mTOR signaling pathway, which promotes apoptosis and inflammation in colonic inflammatory cells [68]. Modulation of this pathway can help to slow down the "inflammation and cancer transformation" and disease progression. The above studies have confirmed the importance of the PI3K/Akt signaling pathway in the treatment of CC, the above results were consistent with the GO enrichment analysis, in which BXD could intervene in CC through inflammatory response modulation and microbial response, while other interventions for CC were consistent with the GO enrichment analysis. Based on the results of the above network pharmacology study, 7 key bioactive ingredients and 14 representative targets were subjected to molecular docking, and most of the compounds could bind well to the proteins, among which the binding energy of Stigmasterol to MMP9 was the lowest, which indicated that the most probable binding site was MMP9.

## CONCLUSION

In summary, the present study was a preliminary exploration of the main chemical components and potential mechanisms of action of BXD for the treatment of CC by network pharmacology and molecular docking using UPLC-Q-TOF-MS/MS method. Our study suggests that naringenin, baicalein, and stigmasterol may be the main active components of BXD, and the protein targets MMP9, TPGS2, and AKT1 may be the potential therapeutic targets of BXD for CC. It may exert therapeutic effects through IL-17, TNF, PI3K-Akt signaling pathway, and cancer pathway.

## STUDY LIMITATIONS

Experimental verification limitations: the data in the study may have limitations regarding sample size and data source comprehensiveness. The databases for predicting targets and pathways could have biases or incomplete information, affecting the accuracy and comprehensiveness of the identified targets and pathways.

Complexity of the disease model: Chronic Colitis is a complex disease influenced by multiple factors in its occurrence, development, and progression. In this study, the disease mechanism might have been simplified to some extent as it focused on merely a few predicted pathways and targets.

Experimental verification limitations: the study relied largely on computational methods like network pharmacology and molecular docking. Although these can predict potential active components and targets, not all of the predicted mechanisms have been experimentally verified.

## AUTHORS’ CONTRIBUTIONS

The authors confirm their contribution to the paper as follows: study conception and design: C.Y., J.W., C.G.; draft manuscript: X.P., R.Z.; Validation: M.W. All authors reviewed the results and approved the final version of the manuscript.

## Figures and Tables

**Fig. (1):**
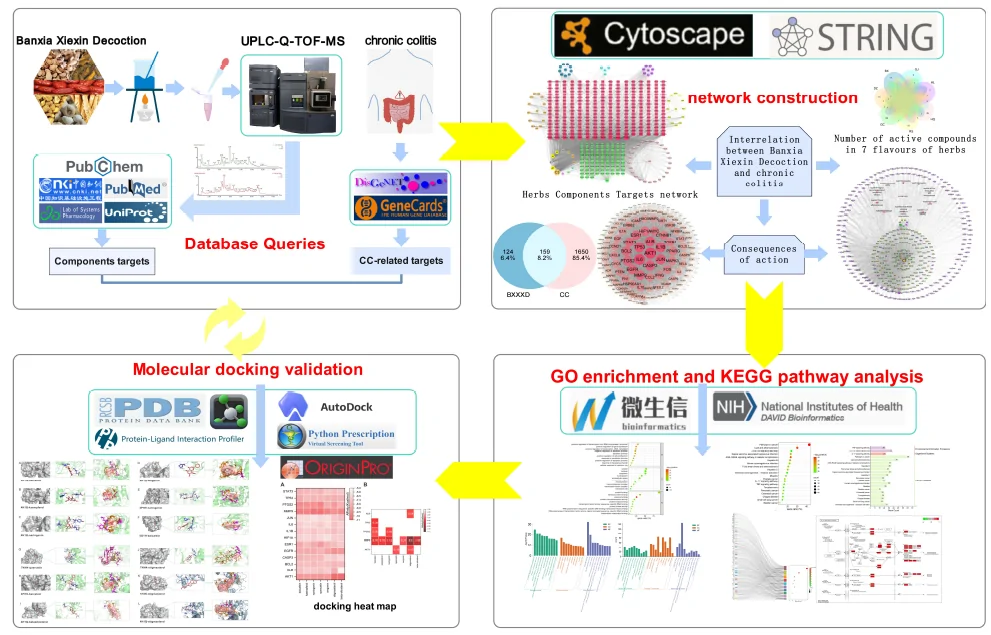
Predictive studies of the mechanism of action of BXD in the treatment of CC.

**Fig. (2):**
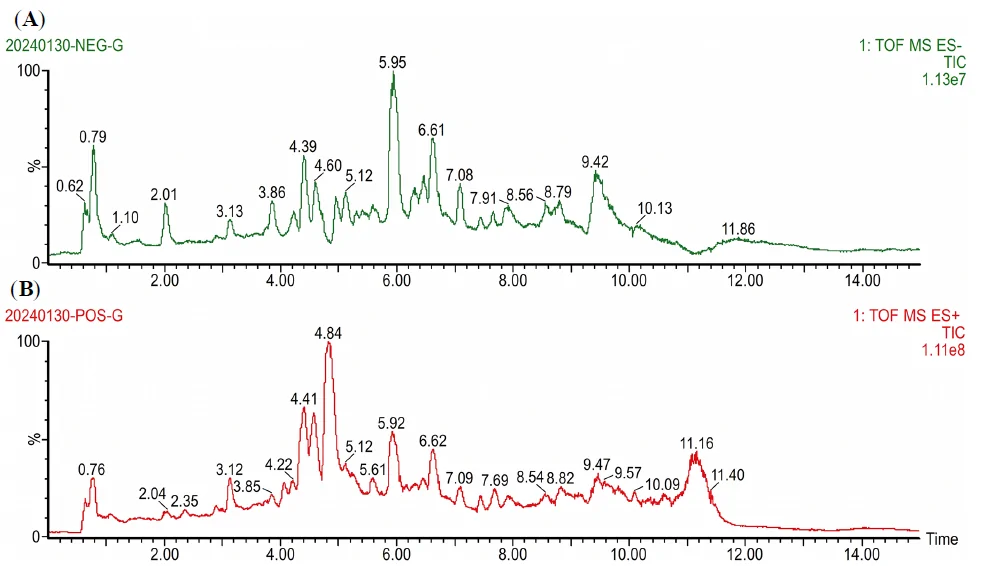
BPI (base peak ion) of Banxia Xiexin decoction from UPLC/Q-TOF-MS analysis. **A**: Negative scan; **B**: Positive scan, The horizontal coordinate is time in min and the vertical coordinate is the electrical signal in mAU.

**Fig. (3):**
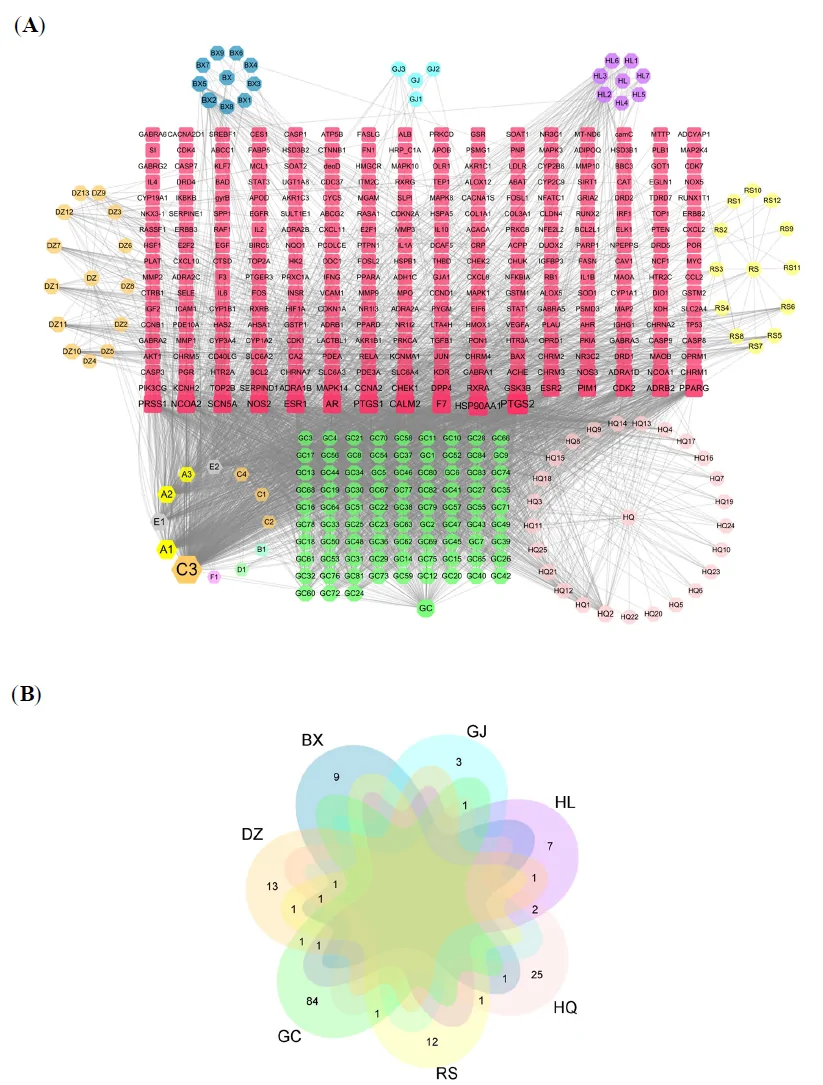
Herb-ingredient-target network diagram with the intersection of active ingredients of 7 herbs. (**A**) Herb-Ingredient-Target network. The labels on the hexagonal node constituent nodes are self-written IDs, and this ID is a correspondence with the Mol ID of the TCMSP database. The ID nodes without serial numbers represent the herbs in the BXD, yellow circle nodes are RS compounds, green circle nodes are GC compounds, dark blue circle nodes are BX compounds, pink circle nodes are HQ compounds, purple circle nodes are HL compounds, orange circle nodes are DZ compounds, light blue circle nodes are GJ compounds, and red square nodes represent predicted targets. Edges represent interactions between compounds and targets, and node size is proportional to the degree of interaction. (**B**) Venn diagram showing the number of active compounds in each flavor of BXD. The purple colour is HL containing 11 compounds, the light green colour is GC containing 88 compounds, the light blue colour is GJ containing 5 compounds, the light yellow color is RS containing 17 compounds, the blue color is BX containing 12 compounds, the light orange color is DZ containing 19 compounds and the light pink color is HQ containing 32 compounds.

**Fig. (4):**
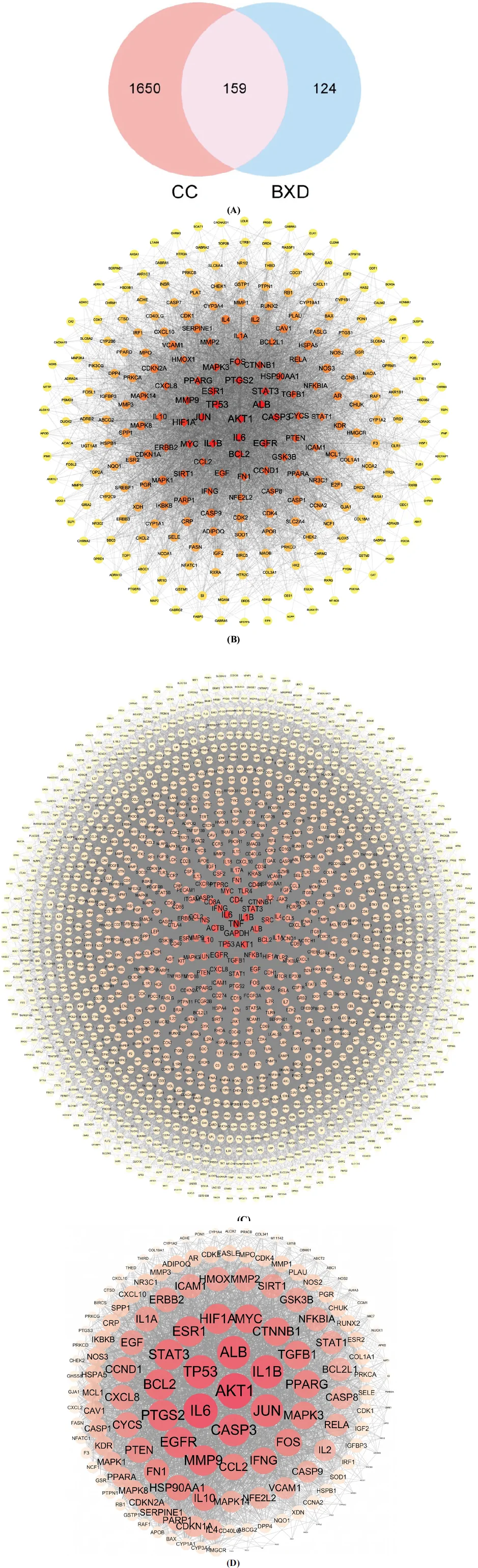
Screening of BXD-CC common targets. (**A**) Venn diagram of targets acting between BXD and CC. (**B**) Network diagram of 269 targets after removing free targets that are not interconnected for 283 targets. (**C**) Network plot of 1504 targets from 1809 targets after removal of free targets that are not interconnected. (**D**) PPI network plot of 159 targets for BXD treated CC derived from the intersection of the 269 component targets that are not free from each other and the 1504 disease targets.

**Fig. (5):**
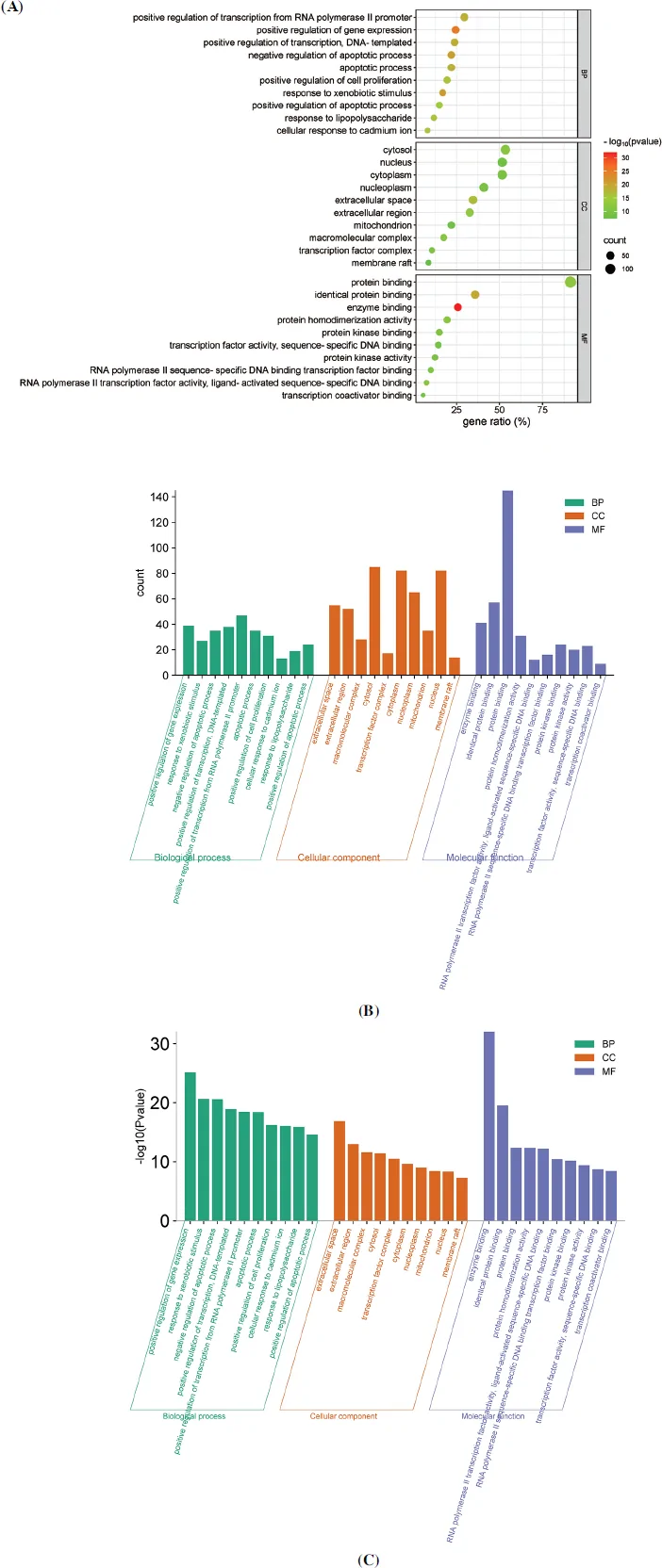
GO functional enrichment analysis. (**A**) Bubble diagram of biological processes, cellular components, and molecular functional category obtained from GO enrichment analysis. The x- and y-axes indicate the gene proportion and full name of the process, respectively, and the color and size of each bubble indicate the *P*-value and the number of genes, respectively; subsequent bubble plots are presented in a similar manner. (**B**) and (**C**) The top 10 biological process (BP) entries, cellular component (CC) entries and molecular function (MF) entries analyzed by GO enrichment are shown as green, orange and purple entries, respectively. (**B**) Y-axis indicates the *P*-value. (**C**) Y-axis indicates the number of genes corresponding to the entry.

**Fig. (6):**
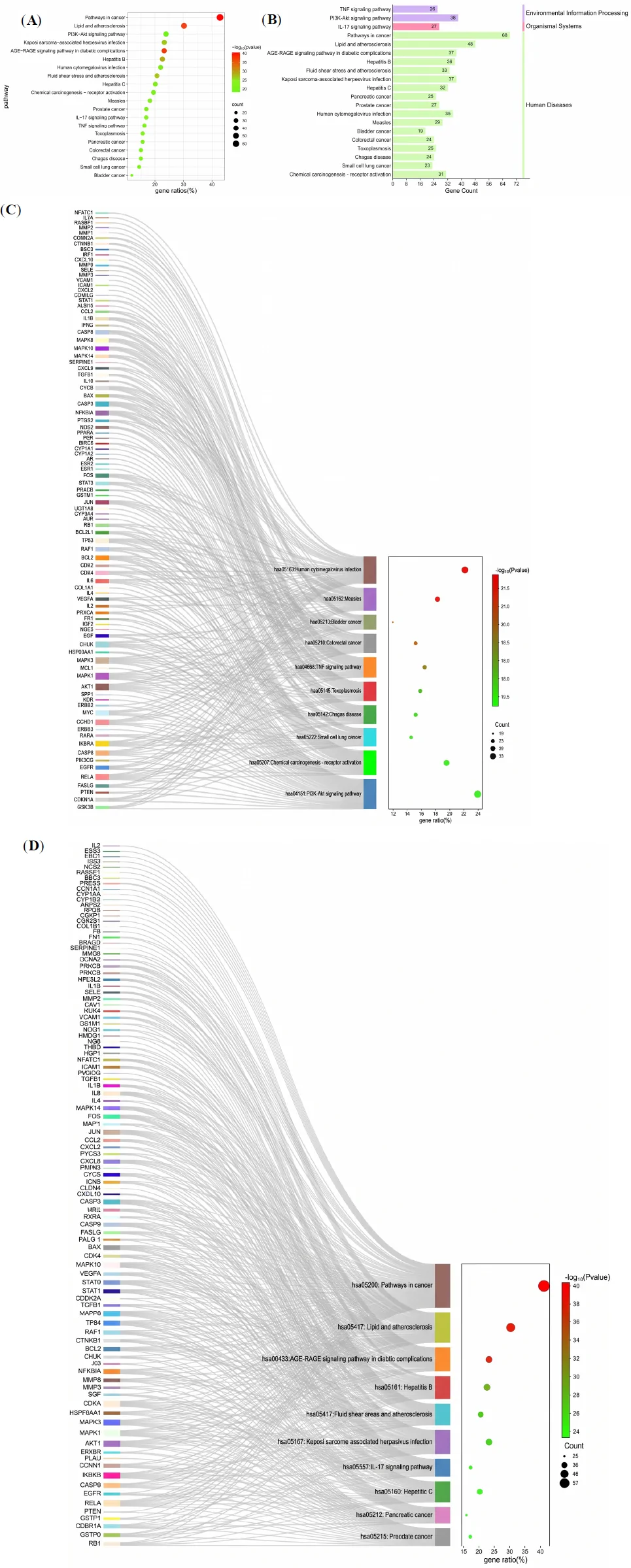
KEGG enrichment analysis. (**A**) Bubble diagram of the top 20 pathways based on KEGG enrichment analysis. (**B**) KEGG types of the top 20 pathways based on KEGG enrichment analysis. (**C**) Sankey diagram of KEGG pathway analysis for BXD treatment of CC therapeutic targets. On the left side of the Sankey diagram, the left rectangular node represents the therapeutic target, the right rectangular node represents the KEGG pathway, and the line represents the attribution of the target and the pathway. On the right side, the top to bottom represents the top 1~10 ranked KEGG pathways, where the bubble diagram explains the information corresponding to the KEGG pathways. (**D**) Same as (**C**) but the right side is from top to bottom representing the top 11~20 ranked KEGG pathways.

**Fig. (7):**
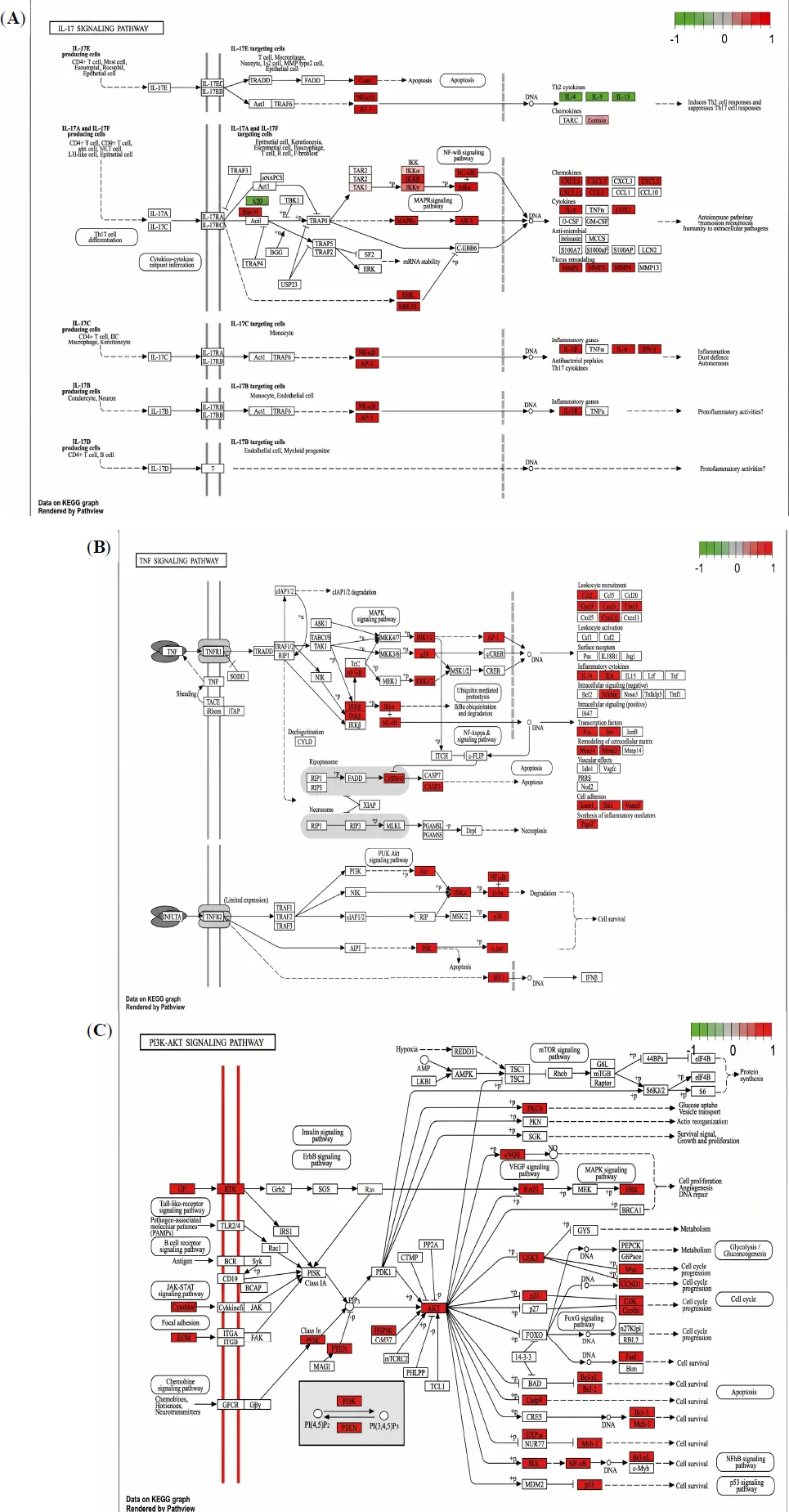
Distribution of key targets in the relevant pathways. (**A**) Distribution of key targets in the IL-17 signaling pathway. (**B**) Distribution of key targets in the TNF signaling pathway. (**C**) Distribution of key targets in the PI3K/AK signaling pathway. Red rectangles represent key targets, and putative targets and genes involved in the pathway-associated genes are shown in red.

**Fig. (8):**
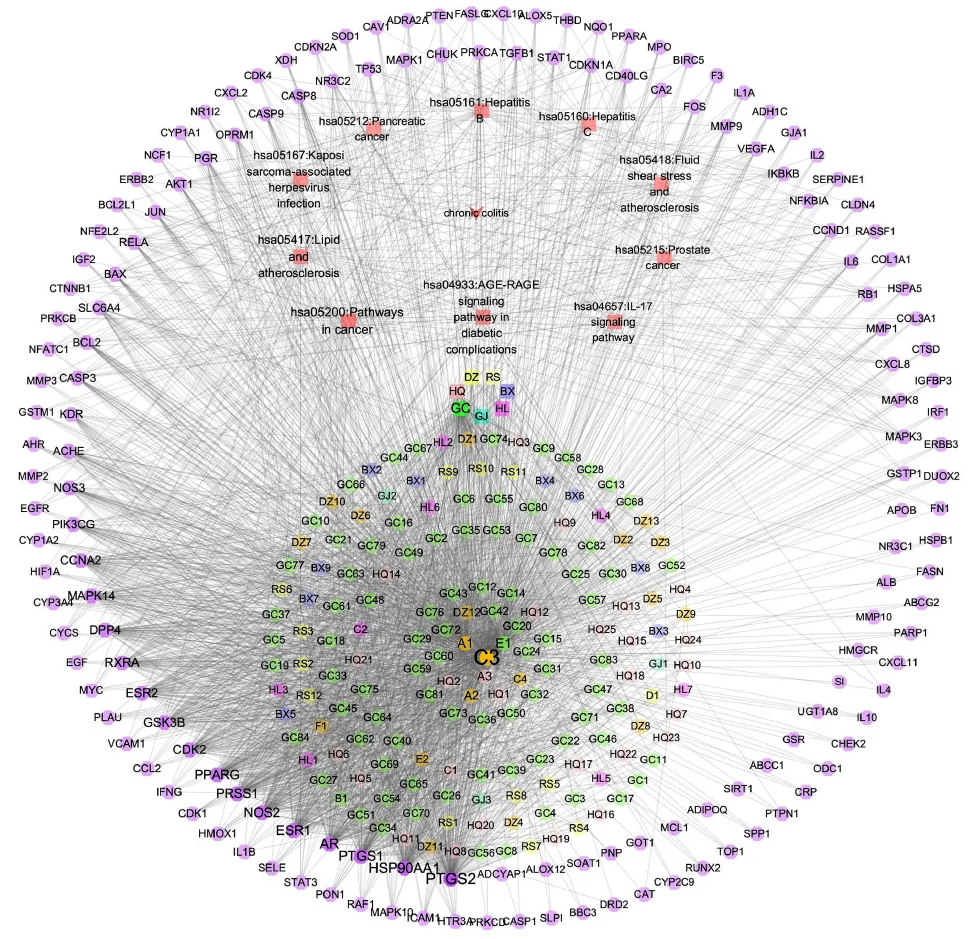
Drug active ingredient-target-of-action-pathway action network. The size of the node text is proportional to the degree of interaction, where the two layers of purple nodes in the outer circle are the targets of action, the red arrow nodes are the diseases, the red squares are the pathways, the light purple nodes are the HL compounds, the pink nodes are the HQ compounds, the blue nodes are the BX compounds, the orange nodes are the DZ compounds, the light yellow nodes are the RS compounds, the green nodes are the GC compounds, and the light blue nodes are the GJ compounds.

**Fig. (9):**
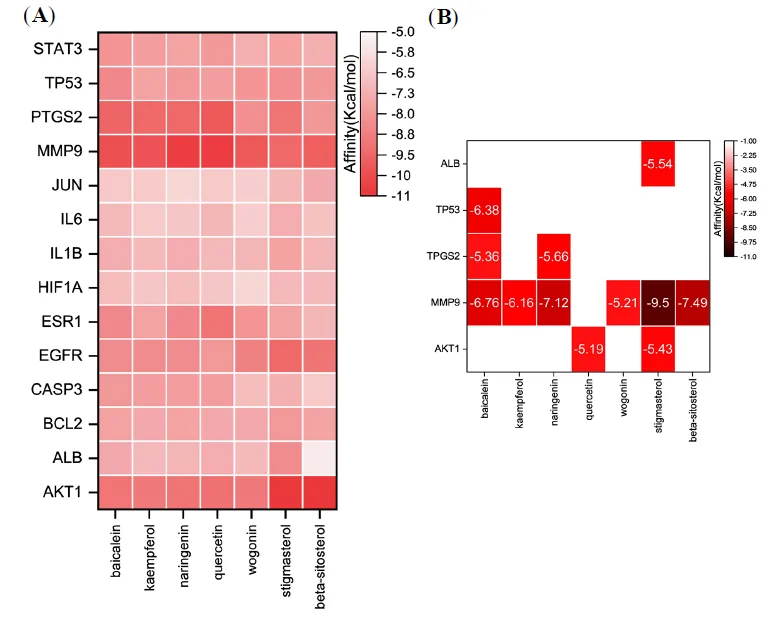
Heat map of active ingredient docking to core target. (**A**) Pre-docking by PyRx software, Y-axis is target and X-axis is compound ligand. (**B)** Binding energies of formation by AutoDockTools 1.5.6 molecular docking, Y-axis is the target, X-axis is the compound ligand.

**Fig. (10):**
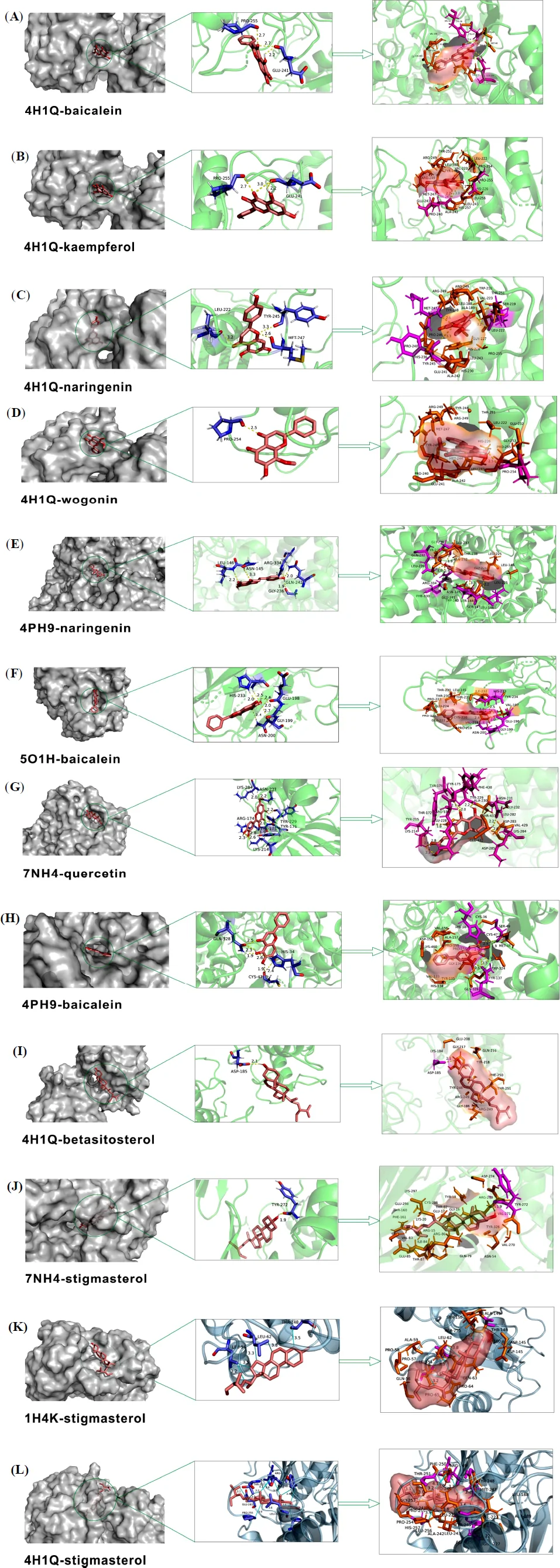
Molecular docking visualization results. (**A-J**) The text in the lower left corner shows the PDB ID corre-sponding to the protein docked to the core component, the left side corresponds to the pocket structure, the middle shows the ligand and re-ceptor binding with hydrogen bonding between them in the active pocket, and the right side shows the 5A residue pocket diagram, in which the effect is indicated by the yellow dashed line, and the text inside the diagram shows the name and distance. (**K,** **L**) Stigmasterol can bind to 7NH4 in the PDB database via hydrogen bonding but exhibits only hydrophobic interactions with 1H4K and 4H1Q receptors in the PDB database without hydrogen bonding.

**Table 1:** Databases, platforms and software used in the research of this paper (Information about the string to search the database, the time of the search, and whether the database is freely available is in the supporting documentation.)

**Pattern**	**Tool**	**Website**
database	TCMSP	https://old.tcmsp-e.com/tcmsp.php
database	HERB	http://herb.ac.cn/
database	STRING	https://string-db.org
database	UniProt	https://www.uniprot.org
database	DisGeNET	https://www.disgenet.org
database	GeneCards	https://www.genecards.org
database	DAVID	https://david.ncifcrf.gov/
database	bioDBnet	https://biodbnet-abcc.ncifcrf.gov/
database	PubChem	https://pubchem.ncbi.nlm.nih.gov/
database	RCSB PDB	https://www.rcsb.org/
platform	Bioinformatics	https://www.bioinformatics.com.cn
platform	PLIP	https://plip-tool.biotec.tu-dresden.de/
software	Cytoscape 3.9.1	https://cytoscape.org/
software	AutoDockTools 1.5.6	https://autodocksuite.scripps.edu/adt/
software	Origin 2021	https://www.originlab.com/
software	OpenBabel2.4.1	https://openbabel.org/
software	PyMol 2.5.0	https://pymol.org/2/
software	PyRx	https://pyrx.sourceforge.io/

**Table 2:** Sources of herbs.

**BXD Raw Herbs**	**Weight**	**Batch**	**The Source**
BX	48.10	J20230601-1	Hebei province
HQ	36.07	J20230601-2	Shanxi province
HL	12.06	J20230601-1	Sichuan province
GJ	36.00	J20230601-1	Yunnan province
GC	36.11	J20230601-1	Inner mongolia province
RS	36.07	J202306001-2	Liaoning province
DZ	41.06	J20230601-1	Xinjiang province

**Table 3:** Elution procedure.

**Time (min)**	**Flow Rate (mL/min)**	**A (%)**	**B (%)**
0	0.200	5	95.0
10.00	0.200	95	5.0
13.00	0.200	5	95.0
15.00	0.200	5	95.0

**Table 4:** MS data acquisition parameters.

**Conditions**	**Negative Mode**	**Positive Mode**
Capillary voltage (kV)	2.5	3.0
Sample cone voltage (V)	40	40
Source offset voltage (V)	80	80
Source temperature (°C)	80	80
Desolvation temperature (°C)	150	150
Cone gas flow rate (L/h)	50	50
Desolvation gas flow rate (L/h)	800	800
Collision energy (V)	6.5	6.5
Ramp collision energy (V)	0~40	0~40
Acquisition mass range (Da)	100-1500	100-1500
MS survey scan time (s)	0.2	0.2

**Table 5:** The same MOL ID corresponds to different Chinese medicines.

**MOL ID**	**TCM**	**ID**
MOL000358	BX, GJ, HQ, RS, DZ	A1
MOL000449	BX, HQ, RS, DZ	A2
MOL002714	BX, HQ	A3
MOL000359	GJ, HQ, GC	B1
MOL002897	HL, HQ	C1
MOL001458	HL, HQ	C2
MOL000098	HL, GC, DZ	C3
MOL001454	HL, DZ	C4
MOL002879	HQ, RS	D1
MOL000422	RS, GC	E1
MOL000787	RS, DZ	E2
MOL000211	GC, DZ	F1

**Table 6:** PPI network construction analysis yielded 14 core targets.

**Gene**	**Protein Name**	**Degree**	**Betweenness Centrality (BC)**	**Closeness Centrality (CC)**
AKT1	RAC-alpha serine/threonine-protein kinase	132	0.03020	0.85870
IL-6	Interleukin-6	128	0.02801	0.83598
TP53	Cellular tumor antigen p53	123	0.02288	0.81865
ALB	Afamin	122	0.03098	0.81443
IL1B	Interleukin-1 beta	122	0.02239	0.81026
JUN	Transcription factor AP-1	120	0.02242	0.80612
CASP3	Caspase-3	118	0.01483	0.79397
EGFR	Epidermal growth factor receptor	113	0.02080	0.77833
MMP9	Matrix metalloproteinase-9	113	0.01602	0.77451
PTGS2	Prostaglandin G/H synthase 2	113	0.02401	0.77833
STAT3	Signal transducer and activator of transcription 3	112	0.01221	0.77451
BCL2	Apoptosis regulator Bcl-2	112	0.01095	0.77451
ESR1	Estrogen receptor	111	0.01910	0.77073
HIF1A	Hypoxia-inducible factor 1-alpha	109	0.00975	0.76329

**Table 7:** Results of KEGG enrichment analysis of key genes (-log10 (P-value) retain the last five decimal places).

**ID**	**Term**	**Count**	**-log_10_(Pvalue)**
hsa05200	Pathways in cancer	68	40.34790
hsa05417	Lipid and atherosclerosis	48	38.53291
hsa04933	AGE-RAGE signaling pathway in diabetic complications	37	37.86566
hsa05161	Hepatitis B	36	28.09756
hsa05418	Fluid shear stress and atherosclerosis	33	26.56238
hsa05167	Kaposi sarcoma-associated herpesvirus infection	37	26.44990
hsa04657	IL-17 signaling pathway	27	23.76879
hsa05160	Hepatitis C	32	23.54062
hsa05212	Pancreatic cancer	25	23.48738
hsa05215	Prostate cancer	27	23.37699
hsa05163	Human cytomegalovirus infection	35	21.88273
hsa05162	Measles	29	21.48017
hsa05219	Bladder cancer	19	20.72584
hsa05210	Colorectal cancer	24	20.64975
hsa04668	TNF signaling pathway	26	20.12843
hsa05145	Toxoplasmosis	25	19.07007
hsa05142	Chagas disease	24	18.81248
hsa05222	Small cell lung cancer	23	18.59688
hsa05207	Chemical carcinogenesis - receptor activation	31	18.41680
hsa04151	PI3K-Akt signaling pathway	38	18.23657

**Table 8:** Topological properties of core components in ingredient-target-pathway networks.

**ID**	**MOL ID**	**Compounds Name**	**Degree**	**BC**	**CC**
C3	MOL000098	quercetin	328	0.26934	0.52381
A1	MOL000358	beta-sitosterol	85	0.02101	0.41840
E1	MOL000422	kaempferol	80	0.04700	0.43219
A3	MOL002714	baicalein	49	0.02486	0.41434
A2	MOL000449	Stigmasterol	36	0.01955	0.40355
HQ2	MOL000173	wogonin	35	0.02927	0.42047
GC60	MOL004328	naringenin	26	0.05502	0.40936
GC72	MOL000392	formononetin	23	0.01026	0.40644
GC20	MOL000497	Licochalcone a	21	0.00779	0.40547
GC42	MOL000354	isorhamnetin	21	0.00893	0.40741
DZ12	MOL002773	beta-carotene	21	0.01859	0.39977

**Table 9:** Pocket binding of ligands corresponding to compounds docked to protein receptors.

**Ligands**	**Targets**	**PDB ID**	**Binding Pockets**
baicalein	MMP9	4H1Q	PRO-255, GLU-241
kaempferol			PRO-255, GLU-241
naringenin			LEU-222, TYR-245, MET-247
wogonin			PRO-254
stigmasterol			ARG-249, THR-251, TYR-248, GLU-241, PRO-255, LEU-222, HIS-226
beta-sitosterol			ASP-185
baicalein	TPGS2	4PH9	GLN-328, HIS-34, CYS-47
naringenin			LEU-146, ASN-145, ARG-334, GLN-242, GLY-236
quercetin	AKT1	7NH4	LYS-284, ASN-231, ARG-174, TYR-229, TYR-176, TYR-175, LYS-214
stigmasterol			TYR-272
baicalein	TP53	5O1H	HIS-233, GLU-198, GLY-199, ASN-200
stigmasterol	ALB	1H4K	THR-148, LEU-62, LEU-54

## Data Availability

The datasets used and/or analyzed during the current study are available from the corresponding author upon reasonable request.

## LIST OF ABBREVIATIONS

AKT: Protein kinase B
BC: Betweenness Centrality
BP: Biological Process
BPI: Base Peak Ion
CC: Cellular Component
CC: Closeness Centrality
COX-2: Cyclooxygenase-2
DC: Degree Centrality
DisGeNET: Disease Gene Network
DL: Drug-Likeness
DSS: Dextran Sulfate Sodium
ESI: Electrospray Ionization
FDR: False Discovery Rate
GO: Gene Ontology
iNOS: Inducible Nitric Oxide Synthase
KEGG: Kyoto Encyclopedia of Genes and Genomes
LE: Liquid Extraction
LPS: Lipopolysaccharide
MF: Molecular Function
MS: Mass Spectrometry
OA: Osteoarthritis
OB: Oral bioavailability
PDB: Protein database
PPI: Protein-Protein Interaction
STRING: Search Tool for the Retrieval of Interacting Genes/Proteins
TCM: Traditional Chinese Medicine
TCMSP: Traditional Chinese Medicine Systems Pharmacology Database and Analysis Platform
UniProt: Universal Protein Resource
UPLC-QTOF-MS/MS: Ultra-high Performance Liquid Chromatography with Quadrupole Time-Of-Flight Mass Spectrometry
