# Supplementary material for: The Potential Mechanisms of Banxia Xiexin Decoction in Treating Chronic Colitis: Insights from UPLC-Q-TOF-MS/MS and Network Pharmacology Studies
Source: Comb Chem High Throughput Screen. 2025 Jul 14;29(16):2318–44. doi: 10.2174/0113862073350796250305225907 (PMC13684976; doi:10.2174/0113862073350796250305225907)
Supplement: Supplementary file 1 [file CCHTS-29-16-2318_SD1.zip › CCHTS-29-16-2318_SD1/Supporting Documents.docx]

**Table 1. Databases, platforms and software used in the research of this paper**

| **pattern** | **tool** | **website** | **string Search** | **retrieval date** | **subscribe** |
| --- | --- | --- | --- | --- | --- |
| database | TCMSP**[27]** | https://old.tcmsp-e.com/tcmsp.php | Banxia, Ganjiang, Huangqin, Huanglian, Renshen, Gancao, Dazao | 2024.03 | Free |
| database | HERB | http://herb.ac.cn/ | Banxia, Ganjiang, Huangqin, Huanglian, Renshen, Gancao, Dazao | 2024.03 | Free |
| database | STRING | https://string-db.org | Protein nam‌es | 2024.05 | Free |
| database | UniProt | https://www.uniprot.org | Protein names | 2024.05 | Free |
| database | DisGeNET | https://www.disgenet.org | Chronic colitis | 2024.04 | Free |
| database | GeneCards | https://www.genecards.org | Chronic colitis | 2024.04 | Free |
| database | DAVID | https://david.ncifcrf.gov/ | 159 intersecting targets | 2024.05 | Free |
| database | bioDBnet | https://biodbnet-abcc.ncifcrf.gov/ | Gene ID | 2024.05 | Free |
| database | PubChem | https://pubchem.ncbi.nlm.nih.gov/ | Banxia Xiexin Decoction, Chronic Colitis, UPLC-Q-TOF-MS/MS, network pharmacology | 2024.02 | Free |
| database | RCSB PDB | https://www.rcsb.org/ | 4H1Q, 4PH9, 7NH4, 5O1H, 1H4K | 2024.05 | Free |
| platform | Bioinformatics | https://www.bioinformatics.com.cn |  |  |  |
| platform | PLIP | https://plip-tool.biotec.tu-dresden.de/ |  |  |  |
| software | Cytoscape 3.9.1 | https://cytoscape.org/ |  |  |  |
| software | AutoDockTools 1.5.6 | https://autodocksuite.scripps.edu/adt/ |  |  |  |
| software | Origin 2021 | https://www.originlab.com/ |  |  |  |
| software | OpenBabel2.4.1 | https://openbabel.org/ |  |  |  |
| software | PyMol 2.5.0 | https://pymol.org/2/ |  |  |  |
| software | PyRx | https://pyrx.sourceforge.io/ |  |  |  |
|  |  |  |  |  |  |
|  |  |  |  |  |  |

**Please note the information from the table:**

Protein nam‌e: In the excel format file of the “protein name”

159 intersecting targets: See table 2.

Table 2. 159 intersecting targets

| PGR | OPRM1 | | ACHE | | NR1I2 | | MAPK8 | | CASP9 | | CYP3A4 | | RUNX2 | |  |
| --- | --- | --- | --- | --- | --- | --- | --- | --- | --- | --- | --- | --- | --- | --- | --- |
| NR3C2 | HSP90AA1 | | EGFR | | THBD | | SLPI | | JUN | | CYP1A2 | | RASSF1 | |  |
| PTGS1 | SLC6A4 | | CCND1 | | SERPINE1 | | CASP1 | | CASP8 | | CAV1 | | CTSD | |  |
| PTGS2 | AR | | BCL2L1 | | COL1A1 | | ADCYAP1 | | PRKCA | | MYC | | IGFBP3 | |  |
| HTR3A | DPP4 | | CDKN1A | | IFNG | | NR3C1 | | TGFB1 | | F3 | | IRF1 | |  |
| RXRA | PIK3CG | | MMP2 | | ALOX5 | | STAT3 | | PON1 | | GJA1 | | ERBB3 | |  |
| VEGFA | RB1 | | ABCG2 | | CAT | | ESR1 | | CCL2 | | MAPK14 | | NFATC1 | |  |
| BCL2 | CD40LG | GSTP1 | | APOB | | PPARG | | SELE | | GSK3B | | PTPN1 | |  | |
| FOS | IL6 | NFE2L2 | | HMGCR | | CA2 | | VCAM1 | | KDR | | MMP1 | |  |  |
| BAX | CDKN2A | NQO1 | | UGT1A8 | | CDK2 | | CXCL8 | | BBC3 | | STAT1 | |  |  |
| MMP9 | NFKBIA | PARP1 | | GSR | | CCNA2 | | PRKCB | | PRKCD | | HSPA5 | |  |  |
| CASP3 | ODC1 | COL3A1 | | ABCC1 | | PNP | | BIRC5 | | FN1 | | ERBB2 | |  |  |
| TP53 | XDH | CXCL11 | | ADIPOQ | | NOS2 | | DUOX2 | | MCL1 | | HMOX1 | |  |  |
| HIF1A | TOP1 | CXCL2 | | GOT1 | | NOS3 | | HSPB1 | | ALOX12 | | PPARA | |  |  |
| CDK1 | RAF1 | CHEK2 | | SOAT1 | | ESR2 | | SI | | CYP2C9 | | CRP | |  |  |
| MPO | SOD1 | CLDN4 | | IL4 | | MMP3 | | IL2 | | IKBKB | | DRD2 | |  |  |
| ALB |  |  | |  | |  | |  | |  | |  | |  |  |

Gene ID: From an excel file named “Gene ID”
